# Probing the Phytochemical Composition and Antioxidant Activity of *Moringa oleifera* under Ideal Germination Conditions

**DOI:** 10.3390/plants12163010

**Published:** 2023-08-21

**Authors:** Axay Bhuker, Anurag Malik, Himani Punia, Craig McGill, Svetla Sofkova-Bobcheva, Virender Singh Mor, Nirmal Singh, Ajaz Ahmad, Sheikh Mansoor

**Affiliations:** 1 Department of Seed Science & Technology, College of Agriculture, CCS Haryana Agricultural University, Hisar 125004, Haryana, India; 2 Department of Agriculture, School of Agriculture, Uttaranchal University, Dehradun 248007, Uttarakhand, India; 3 Department of Biochemistry, College of Basic Sciences & Humanities, CCS Haryana Agricultural University, Hisar 125004, Haryana, India; 4 Department of Sciences, Chandigarh School of Business, Chandigarh Group of Colleges, Jhanjeri 140307, Mohali, India; 5 School of Agriculture and Environment, Massey University, Palmerston North 4442, New Zealand; 6 Department of Clinical Pharmacy, College of Pharmacy, King Saud University, Riyadh 11451, Saudi Arabia; 7 Department of Plant Resources and Environment, Jeju National University, Jeju 63243, Republic of Korea

**Keywords:** antioxidants, biochemical analysis, phenolics, medicinal plant, *Moringa oleifera*, phytochemicals, temperature

## Abstract

*Moringa oleifera* is a rich source of polyphenols whose contents and profile may vary according to environmental conditions, harvest season, and plant tissue. The present study aimed to characterize the profile of phenolic compounds in different tissues of *M. oleifera* grown under different temperatures (25, 30, and 35 °C), using HPLC/MS, as well as their constituent phytochemicals and in vitro antioxidant activities. The in vitro antioxidant activity of the extracts was evaluated using the 2,2-diphenyl-1-picrylhydrazyl (DPPH), 2,2-azino-bis-3-ethylenebenzothiozoline-6-sulfonicacid (ABTS), and ferric-reducing antioxidant power (FRAP) methods. The polyphenolic compounds were mainly found in the leaves at 30 °C. UPLC/QTOF-MS allowed for the identification of 34 polyphenolic components in seedlings, primarily consisting of glucosides, phenols, flavonoids, and methoxy flavones. At 30 °C, the specific activities of antioxidative enzymes were the highest in leaves, followed by seedlings and then seeds. The leaf and seed extracts also exhibited a greater accumulation of proline, glycine betaine, and antioxidants, such as ascorbic acid, and carotenoids, as measured by the inhibition of ROS production. We found that changes in the expression levels of the validated candidate genes *Cu/Zn-SOD*, *APX*, *GPP*, and *TPS* lead to significant differences in the germination rate and biochemical changes. These findings demonstrate that *M. oleifera* plants have high concentrations of phytochemicals and antioxidants, making them an excellent choice for further research to determine their use as health-promoting dietary supplements.

## 1. Introduction

Medicinal plants have been important to human beings for many years thanks to their antioxidant natural healing properties [1]. These result from their contents of bioactive phytochemical compounds, which have demonstrated therapeutic activities against a wide range of neurodegenerative diseases. There is currently much research concerning isolating these bioactive compounds, resulting in the discovery of new drugs of natural origin through modern techniques. Plant extracts are also directly used as alternative therapies against diverse diseases owing to their pharmacological activities, economic viability, and low toxicity compared with synthetic antioxidants, which may have secondary effects on human health [2].

*Moringa oleifera*, commonly known as the drumstick tree or horseradish tree, is a perennial plant that is native to the Himalayan region of Northeastern India, which has a tropical and subtropical climate [3]. It belongs to the Moringaceae family, with the class Magnoliopsida and order Brassicales, and the genus comprises 13 subspecies [4]. India is one of the foremost leading suppliers of the tender fruit of moringa, accounting for around 80% of the global demand, with an annual production of 2.2 million tons [5]. It is native to sub-Himalayan tracts of India, but commercial seeds from warmer areas are available worldwide [6].

The moringa plant has been used for centuries throughout the tropics for medicinal purposes and to improve nutrition, especially in children. Its therapeutic applications include managing blood sugar levels and other chronic diseases related to inflammation and the manifestations of metabolic syndrome. Moringa seeds are used to manage oil and water purification products and are usually chewed for managing diabetes and other ailments. It is well known as an excellent source of nutrition and a natural energy booster. All parts of the plant, including the leaves, flowers, fruits, and immature pods, have nutritional value, making them valuable food sources. Its seeds possess a high content of polyphenolic compounds and fatty acids. Thanks to its medicinal and nutritional properties, the World Health Organization (WHO) has also promoted it as an alternative food source to combat malnutrition [7].

Plants have developed several adaptive strategies in response to abiotic stresses, such as temperature fluctuations, osmotic stress, and dehydration [8,9]. These adaptive alterations include biochemical, physiological, and molecular changes [10]. *Moringa oleifera* is tolerant to dry conditions, where its extensive tuberous root system allows it to survive prolonged periods of drought [11]. The mature and tender leaves of moringa have potent antioxidant activity against free radicals, preventing oxidative damage to major biomolecules and offering significant protection against oxidative damage [12]. Moringa seeds’ biochemical and physiological levels, comprising both primary and secondary metabolites, may also play crucial roles in seed germination, seedling establishment, and plant development [13,14].

Temperature is a climate variable that controls seed biochemical components and plant physiological functions, mainly carbohydrate and phytochemical component biosynthesis [15,16]. The first organ to encounter abiotic stress is the roots, which exhibit a more pronounced inhibitory activity than shoots [17]. The ideal temperature range for moringa is from 20 to above 30 °C [18]. Age-related variations in cell membranes, tissues, and organ responses in *M. oleifera* have been documented [19]. Previously, Muhl [20] reported that a 30/20 °C, rather than 25/15 °C and 20/10 °C, regime is optimal for seedling emergence and post-germination seedling establishment. However, details of the biochemical and physiological processes underpinning seedling establishment under this optimum temperature regime remain speculative. In addition, the effects of temperature fluctuations in various organs and different developmental stages have scarcely been investigated in moringa plants.

In the face of global climate change, moringa is one of the most versatile plants owing to its medicinal and healthy supplement use [21]. As scientific knowledge is constantly being reshaped, additional harmful effects are revealed continuously, and the emphasis has changed to focus on natural products [22]. Moringa has recently attracted increased interest worldwide because of its multi-purpose uses [23]. Despite the significant advantages of moringa, its excessive use in large quantities leads to unfavorable adverse effects because of its anti-nutritional properties [24,25]. Thus, it is imperative to understand the seed germination process concerning the antioxidant system and metabolic activity under optimal germination conditions. Hence, in the present study, we conduct a systematic comparison of phytochemicals in different moringa tissues (leaves, roots, whole seedlings, and seeds) and determine their correlation with diverse biological activities in addition to characterizing their phytochemical accumulation in these other tissues at various temperatures.

## 2. Results and Discussion

### 2.1. Germination Studies

**Figure 1:**
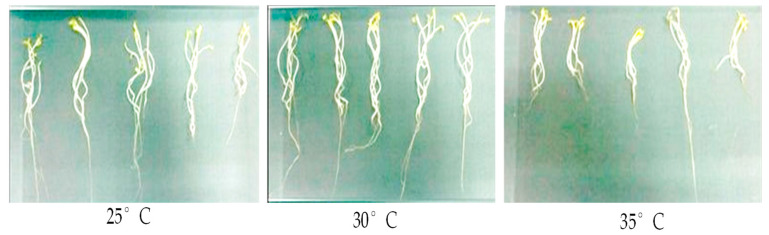
Germinative process of moringa seedlings at different temperatures.

Imbibition, or the seed’s absorption of water, stimulates metabolic activity and the release of chemicals from storage, which causes the embryo to grow and the radicle (or other organs) to penetrate the surrounding tissues [26]. In our study, we observed that the germination rate is significantly affected by temperature. At 30 °C, seed radicle emergence is accelerated and occurs within 48 h, in contrast to 48–72 h at 25 °C and >72 h at 35 °C (Supplementary Figure S1a). In this case, some of the growth metabolites, including carbohydrates, polyols, and enzyme proteins, may be under the control of temperature. With the rising temperature, a considerable decrease in seedling length was observed (*p* < 0.05) (Figure 1) (Supplementary Figure S1b). PKM-1, however, exhibited significantly longer shoot lengths at 30 °C (11.56 cm) than at 25 and 35 °C (7.93 and 3.21 cm, respectively). In the current study, a temperature of 35 °C impacted root length. As the temperature rose, moringa seedling leaves increased in mass, accounting for 48.1 and 73.4 percent of the seeding dry weight (Supplementary Figure S1c). Response surface methodology also indicated that 25 °C and 24 h were the optimal conditions to enhance the accumulation of riboflavin, phenolics, and antioxidant activity of moringa sprouts. In comparison, the total GLS contents were observed at 30 °C for 96 h, and thiamine achieved the maximum content at 30 °C for 24 h [27]. Higher temperatures drastically decreased the PKM-1 seedling vigor. The results show that seedling vigor I and II peaked at around 30 °C (Supplementary Figure S1d,e); in contrast, the seedlings performed poorly at 35 °C. *M. oleifera* showed higher growth under field conditions regarding height, spread, leaf number, leaf area, and leaf dry weight at 25 and 30 °C [28]. The dry weight, root length, and germination index comprise the most important factors to consider when determining temperature tolerance, as these factors determine biomass accumulation [29]. The early seed radicle emergence at 30 °C may be due to rapid hydrolysis and mobilization of the seed reserve due to enzymatic activities, such as the α-amylase conversion of the essential reserve constituent starch into glucose units [1]. On the other hand, seeds that are sensitive to low temperatures during the early stage of imbibition have a lower percentage of germination and are predicted to have poor seedling development and lower plant productivity [14]. The current results align with the finding of Muhl [20], in which the highest germination rates of *Moringa oleifera* were observed at temperatures of 30 °C during both the day and night. At this temperature, the seed may become metabolically active during the germination process, which may be linked to controlling the accumulation of substrates for respiration [19].

### 2.2. Seed Viability Test

**Figure 2:**
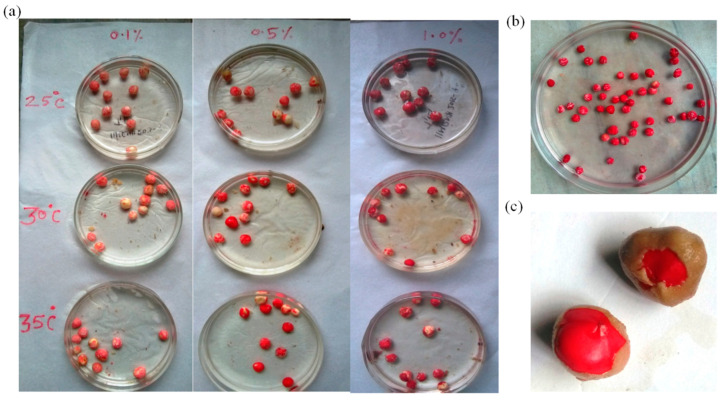
Staining of moringa seed kernels with (**a**) different concentrations of tetrazolium (0.1%, 0.5%, and 1%) under different temperatures; (**b**) stained seed kernels; and (**c**) a close-up showing the unicellular epidermal layer of the inner integument.

A high percentage of viability (>90%) was observed for the moringa seeds used in this study. The results further reveal that both the temperature and concentration of the tetrazolium solution significantly affect seed staining. A light red to dark red color was observed as the concentration of tetrazolium (Tz) solution was increased from 0.1% to 2.0% (Figure 2a). Similarly, the staining also improved with a decrease in temperature. A light red color was visible at a 0.1% concentration of the Tz solution after 45 min (min) at 25 °C and after 30 and 25 min at temperatures of 30 and 35 °C, respectively. At 0.5% Tz concentration, seeds were stained after 20 min; a light pink color was observed at 25 °C, light red at 30 °C, and dark red at 35 °C. At 1.0% concentration, staining was observed after just 15 min at all temperatures (Figure 2b), but was light red at 25 °C and dark red at 30 °C and 35 °C (Figure 2c). The seeds’ most intense color (staining) was observed at 30 °C after 3 h in 1.0% Tz. Pallavi et al. [30] conducted a viability test on moringa seeds and reported observing staining only after 3 h of soaking in 0.5% Tz and only after 6 h in 0.1% Tz. The results indicate that moringa seeds grown at 30 °C have higher viability, based on their more intense red staining than at 25 and 35 °C after 3 h in 1.0% Tz. Kak et al. [31] reported that pre-moistening seeds in 0.1% Tz for six hours at 30 °C or 0.25% Tz for four hours at 40 °C is appropriate for Tz staining of Jatropha. Similarly, Xue et al. [32] also established that 0.5% Tz at 35 °C was optimal for testing pecan seed viability.

### 2.3. Polyphenolic Compounds

The accumulation of polyphenolics in seeds during germination was significantly affected by the assessed temperatures (Table 1). The accumulation of total phenolic content (TPC) was greatly influenced by the temperature, which peaked in leaves at 30 °C (940.73 mg/100 g DW) and stimulated the early emergence of seed radicles (Table 1). Seed phenolic compounds accumulated for seeds at both 25 °C (185.23 mg/100 g DW) and 35 °C (165.28 mg/100 g DW). The highest TPC was found at 30 °C in leaves and then in roots. Similar results were found for flavanols and *o*-hydroxy phenols. The increase in these compounds could also be linked to a plant’s capability to adapt to different temperature regimes, which might result in biochemical compositions that have beneficial synergistic effects. The polyphenols, flavonoids, MDA, and antioxidant capacity in moringa seed at 4 °C, 20 °C, and 30 °C showed that maximum phenolic compounds were observed at 20 °C and 30 °C [33]. da Silva et al. [34] investigated the phenolic composition of different organs of *Moringa oleifera*, such as leaves, hypocotyls, and roots, and reported values ranging between 2.185 ± 0.089 mg GAE/g and 3.805 ± 0.304 mg GAE/g. Similar results were seen in faba beans, where polyphenolics were synthesized and accumulated at approximately the same time as seed priming throughout the germination process [35]. The authors also proposed that mung bean seeds may germinate, affecting phenolic composition, seed bitterness, astringency changes, and seed quality [36]. The bitterness and astringency of plants are influenced by phenolic biosynthesis. During seed germination, the increase in the polyphenolic content of quinoa seeds and their antioxidant activity may confer several health benefits [37]. Likewise, Weidner et al. [38] demonstrated that temperature affects the germination of *Vitis californica* seeds, which have substantial levels of total phenolics and phenolic composition profiles demonstrating high diversity. A recent study on moringa leaves stated that the TPC values obtained for the leaf extracts ranged between 21.7 ± 1.6 mg GAE/g and 39.1 ± 3.3 mg GAE/g, regardless of the extraction conditions used [39]. Besides the already cited factors affecting the polyphenols in moringa, the developmental stage of the different organs analyzed seems to have a crucial effect.

### 2.4. Antioxidant Capacity

The antioxidant capacity of the *M. oleifera* PKM-1 variety was assessed based on the FRAP, DPPH, and ABTS antioxidant activity assays (Table 2). The antioxidant activities were found to be the highest in moringa leaves and lowest in the roots, whole seedlings, and seeds. The highest antioxidant capacity was observed at a temperature of 30 °C, indicated by a lower IC_50_ value, with the values recorded at temperatures of 25 and 35 °C following closely behind. Some of these findings are similar to those of Punia et al. [40], who reported an IC_50_ of 1.87 ± 0.03 for moringa leaves. In more detail, extracts obtained from leaves showed an antioxidant activity around ten times higher than those from cotyledons, independent of the developmental stage and growing substrate of explants. The same trend is confirmed by Santos et al. [41], who tested, through the DPPH radical scavenging activity test, saline and alcoholic extracts obtained from several moringa plant organs (i.e., leaves, rachis, stem, flowers, and seeds), demonstrating that the antioxidant capacity of the extracts markedly depends on the plant organ. The activity of the Fe^3+^/tripyridyl-s-triazine complex decreased in the presence of higher FRAP antioxidant activity [38]. The ABTS and DPPH assays serve to produce nonspecific radicals. At 30 °C, the leaves exhibited the highest increase in antioxidant activity and the seeds exhibited the lowest based on the ABTS assay. The highest germination was observed at 30 °C, which might indicate the relative dominance of several phytochemicals, particularly antioxidants, considering the absence of tannins in *M. oleifera* seeds. The antioxidant activity of polyphenolic compounds is related to their chemical structures, which enable them to function as metal chelators and absorb and neutralize free radicals [42]. The organ dependency of antioxidant activity of moringa extract is also reported by Gómez-Martínez et al. [43], who tested the antioxidant power of moringa leaflets and petioles via the DPPH, ABTS, and FRAP assays. Furthermore, in the endosperm or the cotyledons of the embryo, seeds can accumulate secondary metabolites that will spread in the sprout during its development [44].

### 2.5. LC/ESI/QTOF-MS Identification of Phenolic Compounds in Moringa Seedlings

Information related to the profile of phenolic compounds in moringa seedlings is summarized in Table 3. By comparing the MS and MS/MS spectra from QTOF-MS to data from the accessible mass spectral databases, all phenolic compounds were first identified. These compounds fall under diverse chemical groups: primarily acids, glucosides, saccharides, sterols, esters, phenols, flavonoids, and their derivatives.

Some of the chemicals identified in this study have previously been mentioned in earlier studies. For instance, different portions of moringa contained glucosides. Premi and Sharma [45] employed LC/MS to identify benzoyl-β-D-glucoside, 4-*O*-(6-vanillyl-1)-β-D-glucopyranosyl vanillyl alcohol, and 7-rhamnoglucoside in moringa seed extract. These three compounds possess biological activities such as antioxidant and anti-inflammatory activities. Rodríguez-Pérez et al. [46] identified glucosinolates in moringa leaves. In this study, multiple glucosides were preliminarily identified in ethanol extracts of moringa seeds, e.g., 5,7-dimethoxy-4′-hydroxyflavone-4′-*O*-alpha-L-rhamnose(1-->2)-beta-D-glucoside, nevadensin-7-*O*-[α-L-rhamnosyl(1-->6)]-beta-D-glucoside, taxifolin-3-*O*-glucoside, and glucosinalbin. The UPLC-ESI-QTOF-MS analysis of hydroalcoholic extract allowed for the identification of 24 compounds, with flavonoid derivatives being the most abundant group. Furthermore, the alkaloid trigonelline and sesquiterpenoid abscisic acid were identified for the first time in *M. oleifera* leaves [47]. Leaves of moringa collected from sub-Sahara Africa were analyzed for phenolic components by HPLC–UV–MS. Twelve flavonoids were identified, including quercetin, kaempferol glucosides, and glucoside malonates as major constituents [48]. LC–MS analysis under the optimum conditions led to the identification of 12 bioactive compounds. The major compounds were identified as 1*H*-pyrazol-4-yl methanol, 1-(2-methyl-5-nitro-1*H*-imidazol-1-yl) propan-2-ol, 1,4-naphthoquinone, luteolin-6-*C*-glucoside, and hesperidin, which possess different biological activities [45].

As expected, phenolics, flavonoids, and their derivatives are abundant in *M. oleifera* seeds, and 4′,5,6,7-tetramethoxy-flavone, chelidimerine, maltose, 5,7-dimethoxy-4′-hydroxyflavone-4′-*O*-α-L-rhamnose(1-->2)-beta-D-glucoside, and (2*R*,3*R*)-3,5,7,2′,6′-pentahydroxyvflavanone were the main compounds in the seed extract. Moreover, several studies suggested that moringa is a good source of bioactive compounds such as flavonoids, phenolics, and their derivatives [45,46,49].

### 2.6. Polyamines

The content of total polyamines remains constant during plant development; however, they accumulate under stressful circumstances (Supplementary Figure S2). A chromatogram of benzoylated polyamine standards was obtained by reverse phase HPLC using a C18 column for separation (Supplementary Figure S3a). The peak identities were verified by injecting polyamine references into the plant tissue extract (Supplementary Figure S3b). The retention time of each peak, expressed in min, is accurate and consistently reproducible. On the left side of the chromatogram are peaks corresponding to major byproducts of the benzoyl reaction that were eluted and separated from benzoylated polyamines. Putrescine (Put), spermidine (Spd), and spermine (Spm) are the three naturally occurring amines (Mol. wt. 202.34). The increased concentration of a range of polyamines in moringa leaf tissues suggests the occurrence of ROS scavenging in these tissues [9,50,51]. Each peak on the chromatogram represents 0.05 mM of a specific amine. Table 4 displays the accumulated amounts of several polyamines at early developmental stages.

Table 4 shows the variations in polyamine profiles. Spermine (Spm) accumulates more rapidly at higher temperatures. However, the basal level was raised to 30 °C. Spm accumulation was not more significant at 35 °C than at 30 °C. Roots and seedlings also showed a comparable rise in Spm concentration with rising temperature levels. At all temperatures, the amount of endogenous spermidine (Spd) in the organism gradually increased over time as they developed, whereas the amount of Spd in the seedlings decreased. As a result, the average Spd content was higher at 30 °C than at 35 °C. Putrescine (Put) accumulated similarly to Spm and increased.

### 2.7. Antioxidative Enzymes

**Figure 3:**
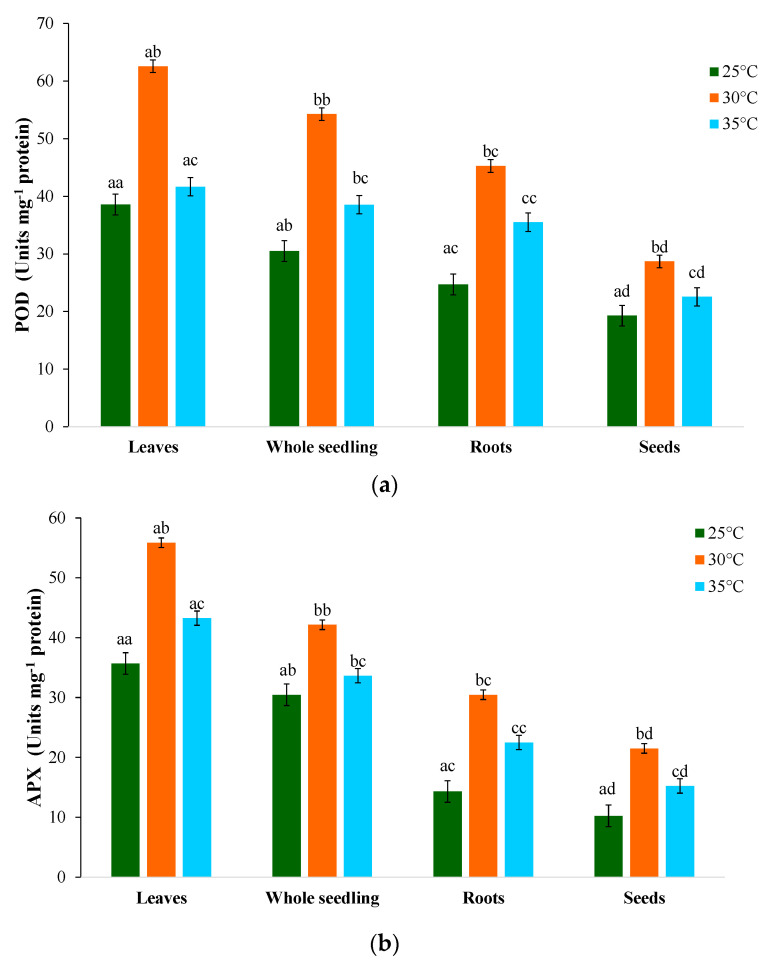

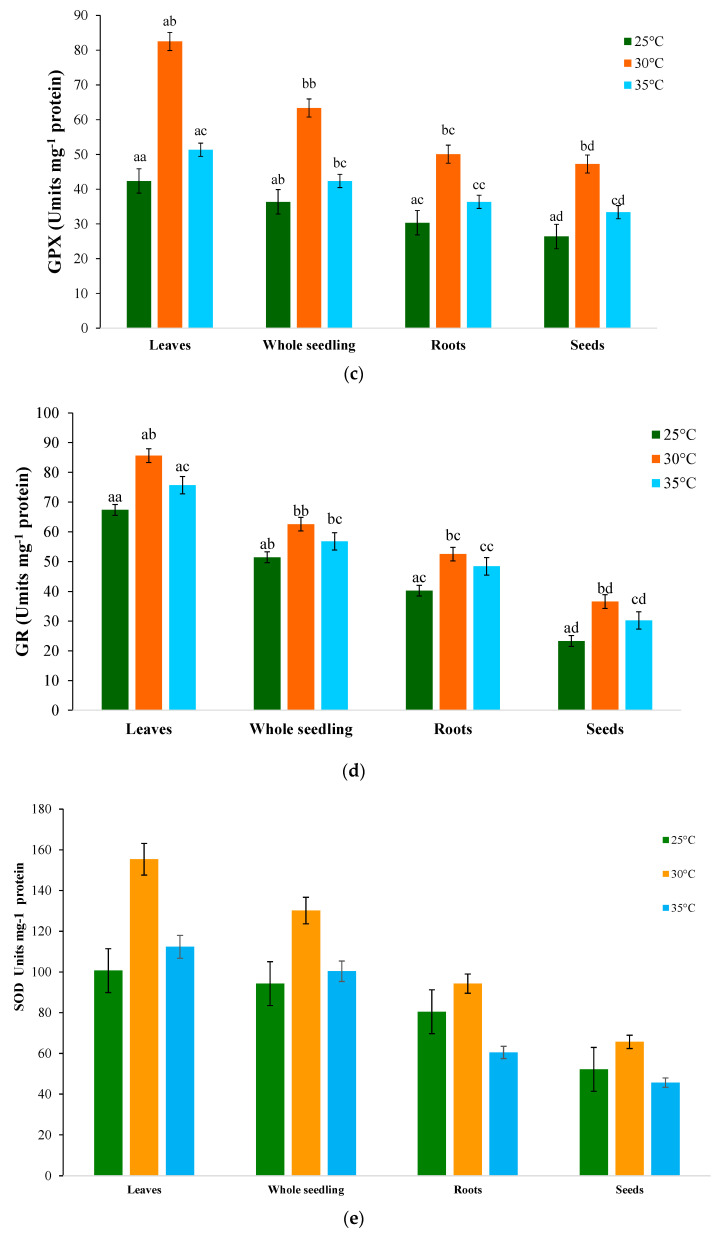

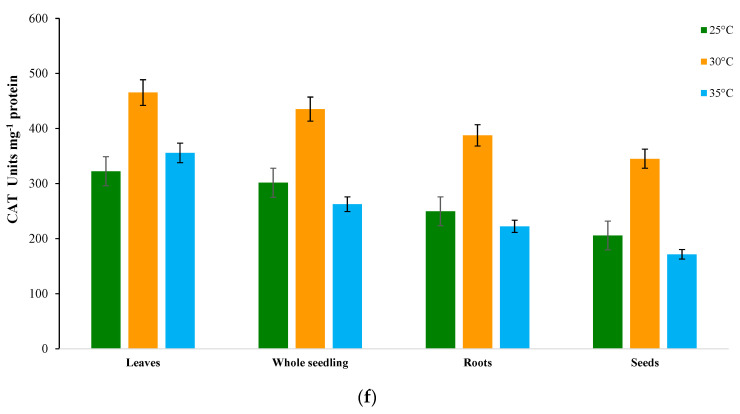
Effect of different temperatures on the activities of (**a**) superoxide dismutase (SOD); (**b**) catalase (CAT); (**c**) peroxidase (POX); (**d**) ascorbate peroxidase (APX); (**e**) glutathione peroxidase (GPX); and (**f**) glutathione reductase (GR) of *M. oleifera* PKM-1 variety. Data represent the mean ± standard deviation of three independent experiments. Different superscript letters within a column indicate significant differences (two-way ANOVA, post hoc Tukey’s test; *p* ≤ 0.05).

The activity of superoxide dismutase (SOD) in seeds was significantly influenced by temperature. SOD showed an upward trend for seed germination at 30 °C; the activity was elevated for leaves (46.2 percent) as it encounters a high cell metabolic increase (Figure 3a). On the contrary, for the other two temperatures, 25 °C and 35 °C, there was a rise in SOD activity but less activity than at 30 °C (27.3% and 21.4%, respectively). The essential critical function of SOD is to catalyze the dismutation of superoxides into H_2_O_2_; an increase in this ROS product may be related to enhanced SOD activity. This further demonstrates the strong relationship between SOD and CAT activity, as the latter is dependent on the level of H_2_O_2_ produced by SOD [52]. The regulation of steady-state ROS formation by SOD is a crucial mechanism for coping with cell oxidative stress because O_2_ serves as a precursor to more toxic or highly reactive ROS [53]. Hence, our results indicate that a temperature rise significantly affects the SOD activity, which reaches its maximum at 30 °C. Earlier results suggest that elevated SOD activity and cytoplasmic ROS levels affect numerous plant vital processes, including developmental changes such as seed germination [54]. The SOD mainly dismutases the superoxides into H_2_O_2_; the higher product of this ROS might be associated with increased SOD activity. This also confirms the holistic strength between SOD activity and CAT activity; the CAT activity depends on the H_2_O_2_ concentration, which is the product of SOD [52]. Higher ROS generation may result in increased SOD activity, or seeds may use it to defend against oxidative damage. Early reports illustrate that increased SOD activities and cellular ROS levels were involved in the life of many plants, including developmental courses such as seed germination [55].

The temperature had a significant effect on catalase (CAT) activity, which was elevated during seed imbibition, showing a steady upward trend (52.1%), and subsequently decreasing at 30 °C (Figure 3b). The CAT activity at the other two temperatures was approximately constant across the measured seed and seedling components. Leaves showed the highest CAT activity, followed by whole seedlings and roots, whereas intact seeds showed the least CAT activity. Owing to the high seed metabolic rate, the enzyme activity may have risen in response to the increased H_2_O_2_ accumulation. Therefore, the cumulative impact of low molecular antioxidants generated during seed imbibition may influence seed germination [56]. As it reduces H_2_O_2_ generated during the β-oxidation of fatty acids, CAT is necessary for oily seeds and plays a significant role in the early stages of seedling development [57]. Because the quantity of oxidants in cells is correlated with the level of CAT present, increased CAT activity may indicate cellular ROS generation [58,59]. Transgenic tobacco plants overexpressing *Cu/Zn-SOD* exhibit temperature and salt stress resistance. The accumulation of H_2_O_2_ starts catalase and appears to be consistently effective in eliminating increased H_2_O_2_ levels [60]. Seed germination could thus be stimulated in response to the cumulative effect of low molecular antioxidants produced during seed imbibition time. In oily seeds, CAT is required and contributes hugely to the early events of seedling growth because it alleviates H_2_O_2_ produced during β-oxidation of the fatty acids [61]. Increased CAT activity could indicate the cellular evaluated ROS because the amount of CAT present resembles the level of oxidants at the cellular level [58].

An increase in peroxidase (POD) activity was observed at different temperature regimes (Figure 3c). In leaves, POD activity increased by 57.3% at 30 °C and 31.2% at 35 °C. Similar basal levels of POD activity was also found in the tissues of the roots under varying temperature regimes. The POD activity in seeds showed a similar increase, with values approaching approximately 3.6-fold at 30 °C and 2.5-fold at 35 °C. However, in the PKM-1 variety, the percentage increase in POD activity was lower in whole seedlings and seeds than in leaves. POD enzymes may play a vital role in rapidly eliminating H_2_O_2_ in stress-tolerant genotypes. Under stress, *A. lividus* showed a significant decrease in POD activity and increased heat shock proteins. The maximum elevation was observed at 30 °C and the minimum at 25 °C. When leaves were exposed to high temperatures, lower SOD, CAT, and POD activities were seen owing to the disruption of optimal conditions; these activities may be related to a range of metabolic activities [62]. An increase in peroxidase activity has been linked to cell wall resistance from lipid peroxidation, cross-linking, and lignification under various abiotic stresses [63].

In leaves, the ascorbate peroxidase (APX) activity increased with the rise in temperature, peaking at 30 °C (64.8%) and declining at 35 °C (23.7%) (Figure 3d), with the optimal activity at 25 °C (34.3%). Roots showed a similar pattern to leaves, but the total leaf activity was higher. The same trend was observed in seeds and whole seedlings, but APX activity was higher in leaves overall. A considerable increase in glutathione peroxidase (GPX) activity (U mg protein^−1^) was demonstrated, with differential responses (Figure 3e). At 30 °C, an increase in GPX activity was recorded in leaf tissue (56.8%), while the activity was lower at 35 °C (32.1%) and lowest at 25 °C (23.9%). Roots, followed by whole seedlings and seeds, exhibited a similar trend of an increase in GPX activity. Plant peroxidases, namely GPX and APX, use ascorbate (reduced form) as an electron donor during the first cycle of the ascorbate–glutathione cycle to sequester chloroplastic H_2_O_2_ [64]. The increase in temperature led to increased glutathione reductase (GR) activity in all sample tissues (Figure 3f), which was higher in leaves. The GR activity increased by 52.6% at 30 °C, while the activity declined to 32.6% at 35 °C and was lowest at 25 °C (21.6%). At the seedling growth, the GR activity increased in roots by 28.9%, 48.2%, and 33.8% at 25, 30, and 35 °C, respectively. Our findings were also in line with reports of Dučić et al. [65], symptomatic of the participation of SOD, POD, GPX, and GR in the defense mechanism during germination and early seedling development. At varying temperatures, roots showed a similar pattern, but the GR activity was lower in roots than in leaf tissue, whole seedlings, and seeds [66]. GR is necessary for glutathione replenishment in ascorbate–glutathione reactions in an NADPH-dependent process. In *Macrotyloma uniflorum* [67], the GR activity was reported to be more upregulated in tolerant cultivars than in susceptible varieties, indicating the potential role of GR in the abiotic stress response during the early seedling growth stage.

### 2.8. Non-Enzymatic Antioxidants

**Figure 4:**
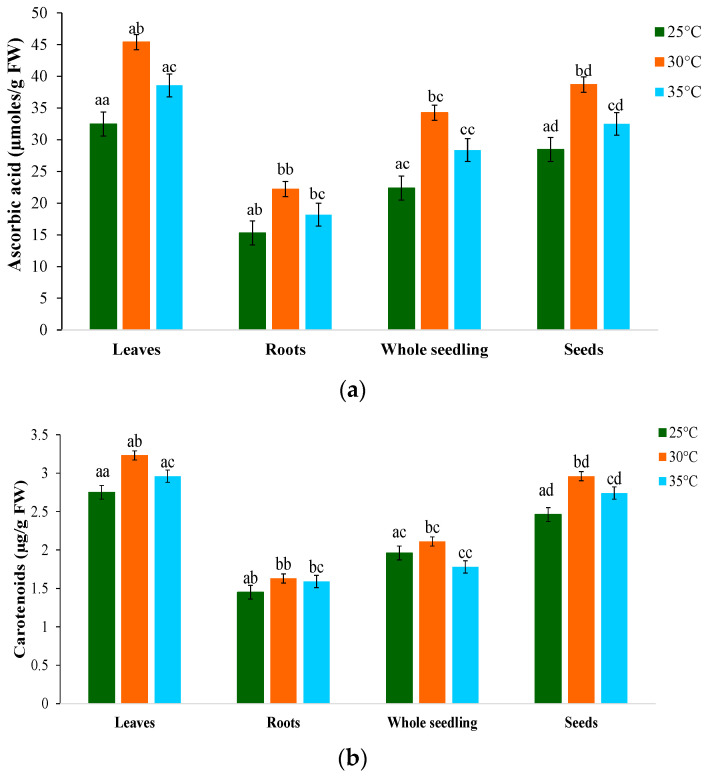
Effect of temperature on (**a**) ascorbic acid and (**b**) carotenoid contents of *M. oleifera* PKM-1 variety. Data represent the mean ± standard deviation of three independent experiments. Different superscript letters within a column indicate significant differences (two-way ANOVA, post hoc Tukey’s test; *p* ≤ 0.05).

Ascorbic acid and carotenoids are non-enzymatic antioxidants [58,60]. Ascorbic acid (ASC) accumulated significantly when the temperature increased (Figure 4a). ASC content increased by 53.6% at 30 °C compared with only a 25.8% increase at 35 °C in leaves, as ascorbate is a reducing equivalent in the ascorbate–glutathione cycle. The oxidation and subsequent regeneration describe the biological activity of ascorbate in its reduced form. Under increasing temperature conditions, a significant decline in carotenoid content was observed, with a greater decrease in roots followed by whole seedlings and seeds (Figure 4b). A higher percent of reduction was recorded at 35 °C, i.e., 42.3% and 51.9% for whole seedlings and seeds, respectively. Temperature resulted in a significant increase in the carotenoid content in leaves. Compared with 35 °C, where the percentage increase was only 21.3%, the carotenoid concentration was higher at 30 °C (55.2%). The increased temperature significantly affected carotenoid content, which dropped by 42.1 percent in roots, 32.6 percent in whole seedlings, and 26.8 percent in seeds. The carotenoid levels were not higher in roots than in leaves. The ASA–GSH cycle may be essential for neutralizing free radicals based on increased levels of ASA and GSH and their reduced redox states [68]. According to several studies, concentrations of photosynthetic pigments, such as carotenoids, decrease in response to abiotic stress exposure owing to disruption of the photosynthetic machinery [69,70,71,72].

### 2.9. Compatible Solutes

**Figure 5:**
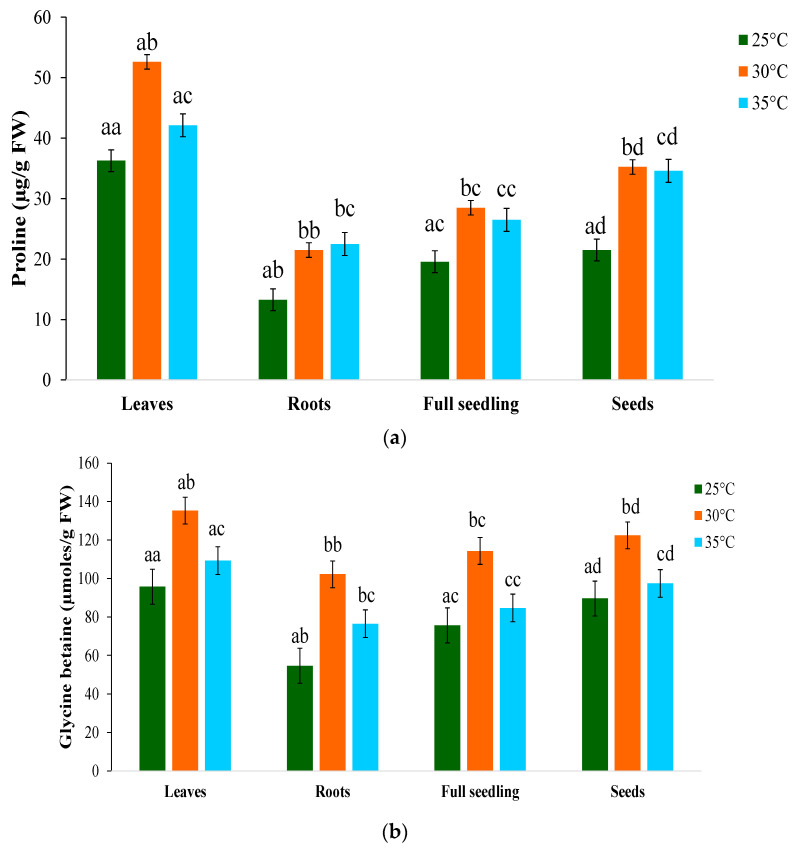
Effect of temperature on (**a**) proline and (**b**) glycine betaine contents of *M. oleifera* PKM-1 variety. Data represent the mean ± standard deviation of three independent replicates. Different superscript letters within a column indicate significant differences (two-way ANOVA, post hoc Tukey’s test; *p* ≤ 0.05).

Proline (Figure 5a) and glycine betaine (Figure 5b), which are compatible solutes whose levels increase significantly in leaves and roots under temperature stress, were found to be affected by temperature. The percentage increase in proline was at its highest at 30 °C (51.2%) and at its lowest at 35 °C (23.6%) compared with 25 °C. Proline activity was higher at 30 °C and lower at 35 °C compared with the optimal temperature. Additionally, the proline concentration increased in the early stages of seed germination before declining as the seed radicle emerged. Organic osmolytes’ accumulation effectively contributed to osmotic adjustments and soluble sugar, maintaining turgidity and protecting cellular metabolism from salt toxicity [73]. The accumulation of both proline and soluble sugar contents indicates their active involvement in osmotic adjustment and temperature tolerance of moringa, as reported in different plant species [74,75,76]. Plants accumulate osmolytes, including polyamines, glycine betaine (GB), proline, and proteins, to maintain turgor pressure and osmoregulatory [77] mechanisms in response to abiotic stressors, which reduce the cytoplasmic osmotic potential and promote water absorption. Glycine betaine exhibited the highest percentage increases in leaves at 30 °C, whereas it increased by 22.5 percent at 35 °C. At all temperatures, glycine betaine levels increased significantly in roots, whereas the accumulation was lower in leaves. Higher glycine betaine concentrations were found in seeds germinated at temperatures below 30 °C, which suggests that this temperature regime is unfavorable for growth, resulting in the regulation of defensive mechanisms to cope with stress to allow seed germination to continue [78].

Nevertheless, post-seedling development might be influenced by delayed morphological advancement. As an osmoregulatory solute, glycine betaine (GB) is naturally synthesized in plants, particularly in the leaves [79]. When chloroplasts are exposed to high-stress conditions, GB can preserve the O_2_-generating apparatus [71]. Free proline accumulation in higher plants is a well-documented response to various abiotic stressors [80]. Enhanced production and reduced breakdown of proline and glycine betaine may result in an increased accumulation of these components under stress environments.

### 2.10. Sugar Mobilization

**Figure 6:**
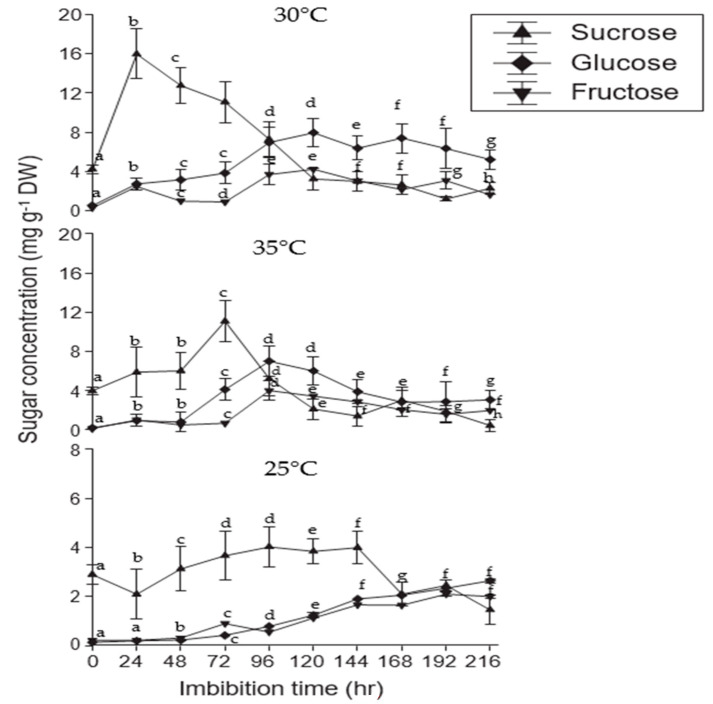
Impact of different temperature conditions (25, 30, and 35 °C) on carbohydrate accumulation in moringa seeds during germination. The vertical bars represent the mean ± standard deviation of three independent experiments. Different superscript letters within a column indicate significant differences (two-way ANOVA, post hoc Tukey’s test, *p* ≤ 0.05).

Seed germination at 30 °C resulted in a rapid and dramatic decrease in sucrose content (Figure 6). When exposed to various temperature regimes, the number of hexoses found in seeds, such as sucrose, glucose, and fructose, varied significantly during the germination process. Sucrose concentrations reached a maximum of 30 °C within 24 h (14.9 3.7 mg/g DW), while they exhibited a significant reduction at 35 °C (3.0 2.1 mg/g DW). At 30 °C, glucose and fructose levels increased and then declined as seedlings began to emerge. A similar carbohydrate pattern was also observed for the two temperatures of 35 and 25 °C as that found at 30 °C: sucrose was shown to be a dominant sugar at all temperature regimes. Seed glucose and fructose gradually declined at 35 °C, whereas there was an increase in both sugars at 30 °C. Safflower seeds had maximum sucrose concentrations of 13.11.9 mg/g dry weight (DW) at 30 °C and 3.2% or 1.5 mg/g (DW) at 35 °C.

Additionally, these compounds engaged in stress signaling and modulating gene expression under stressful conditions [81]. Soluble carbohydrates function as osmoprotectants inside the cell [82], so elevations of their levels in moringa augment the ability of the plant to withstand a wide range of stresses. These compounds protect and stabilize enzymes and proteins, reduce the oxidation of lipid bilayers, work as free radical scavengers and cell redox balancers, provide sites for carbon and nitrogen storage, and are involved in cytosolic pH regulation. Exposure of sorghum embryos to drought or salt stress has resulted in enhanced sugar levels, which may assist in osmoregulation under stressful conditions [37,49,59].

### 2.11. Gene Expression Analysis

**Figure 7:**
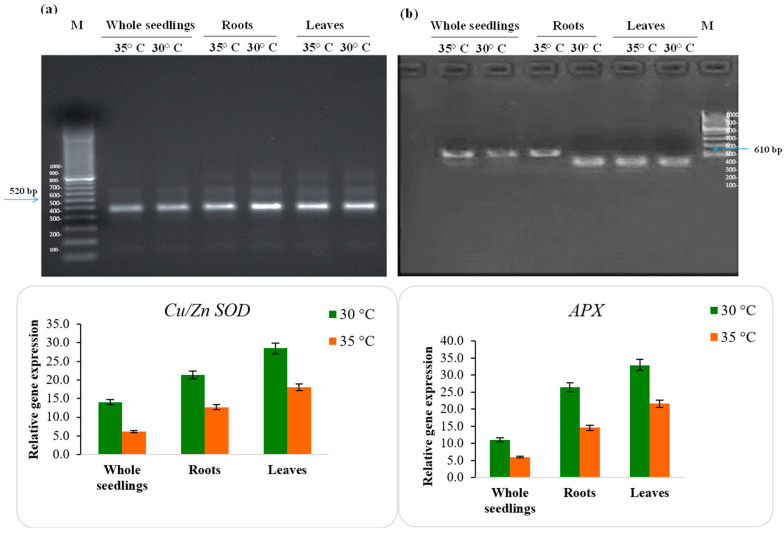

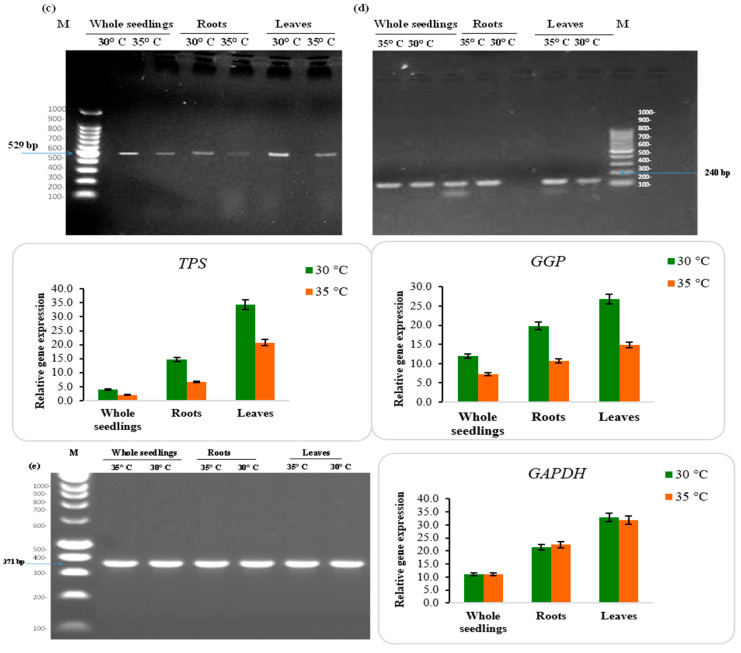
Semi-quantitative analysis of candidate genes in moringa. (**a**) Cu/Zn superoxide dismutase 1 (*Cu/Zn-SOD*); (**b**) ascorbate peroxidase (*APX*); (**c**) trehalose-6-phosphate synthase (*TPS*); (**d**) GDP-L-galactose phosphorylase (*GGP*); and (**e**) glyceraldehyde-3-phosphate dehydrogenase (*GAPDH*) for normalization based on constitutive expression under different temperature regimes. M: molecular marker. Statistical difference was calculated by standard deviation.

Semi-quantitative RT-qPCR was performed to characterize the expression of moringa genes based on relative amplification. Densitometry was conducted to quantify and interpret semi-quantitative gels, and the relative intensity differences between different salt concentrations compared with the control were evaluated. The PCR products were prepared using specific primers for the genes *Cu/Zn-SOD*, *APX*, *GGP*, and *TPS,* resulting in a single band (Figure 7). Their upregulation with elevated temperature demonstrates that the cellular defensive strategy involves combating the detrimental impact of the temperature inside the cytosol. At 30 °C, the higher expression of *Cu/Zn-SOD* (Figure 7a) and *APX* (Figure 7b) indicates resistance to the detrimental effects of temperature.

In contrast, the expression of these genes was reduced at 35 °C. The expression levels peaked at 30 °C and declined at 35 °C. *GAPDH* was constitutively and stably expressed under the different temperature regimes (Figure 7e). The higher expression of these genes at 30 °C indicates that PKM-1 might be exploited as a temperature-resistant variety owing to its superior genetic characteristics. Trehalose-6-phosphate (*TPS*) acts as a specific signal of sucrose status and a regulator to modulate carbon metabolism within the plant. Quantitative real-time PCR analysis showed that the expression patterns of *TPS*s displayed group specificities in *M. oleifera*. *MoTPS* genes were closely related to reproductive development and Group II *MoTPS* genes were closely related to high-temperature resistance in leaves, stem, stem tip, and roots [83]. Zhang et al. [84] studied the expression of *APX*, *GGP*, and *TPS* genes under different abiotic stresses, such as drought, salinity, heat, and cold, and their results indicated that each gene was differentially upregulated, which might show their varying roles in response to different abiotic stress. Punia et al. [72] reported that the concomitant upregulation of *Cu/Zn-SOD*, *APX*, and *GPX* genes in sorghum genotypes is suggestive of tolerance under stress conditions [85]. Deng et al. [86] aimed to establish reliable reference genes for moringa via RT-qPCR analysis of gene expression and observed that, out of eighteen candidate reference genes, only ribosomal protein L1 (*RPL1*) and acyl carrier protein 2 (*ACP2*) could be considered suitable reference genes in all tested samples. Identifying these potential genes may provide a molecular basis for identifying temperature-resistant moringa cultivars.

## 3. Materials and Methods

### 3.1. Plant Material

**Figure 8:**
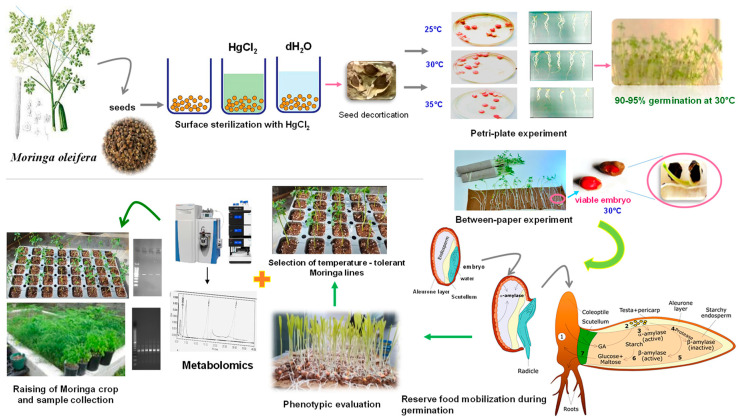
Outline of the experiment. *M. oleifera* PKM-1 variety germinated and grown under varying regimes temperatures (25, 30, and 35 °C) was phenotypically evaluated. Leaves, roots, whole seedlings, and seeds from three random seedlings were pooled, treated as one biological replicate, and used in the metabolic analysis. HPLC was performed to assess the concentration of polyamines at varying temperatures. Total RNA was extracted from different tissues and the expression level of candidate genes was evaluated.

This study was conducted at the Department of Seed Science and Technology, CCS Haryana Agricultural University, Hisar, India, from 2019 to 2020 and 2020 to 2021. The *M. oleifera* [PKM 1] seeds were obtained from the Department of Vegetable Crops, Tamil Nadu Agricultural University, Coimbatore, India. To assess the impact of temperature on moringa seedlings, experiments were conducted in the laboratory at different temperature regimes (25, 30, and 35 °C) (Figure 8). Three biological replicates of each treatment were used.

### 3.2. Seedling Establishment

Moringa seeds were chosen based on their color and size. Before sowing at a depth of 5 cm in silica soil, the seeds were washed with distilled water, air-dried, and then surface-sterilized with 0.01% mercuric chloride (HgCl_2_) solution to prevent fungal infection. After seedling emergence, the seeds were thinned to a maximum of five seedlings per tray. After 15–20 days, the seedlings were assessed as healthy; second leaf growth was observed; and different parts of the seedling, i.e., whole seedling, seedling root, and seedling shoot, were collected separately. Then, the seed components were stored at −80 °C until use in further molecular and biochemical analysis. After eight weeks, the experiment was terminated and a statistical analysis of the gathered data was conducted to evaluate the impact of temperature on the mobilization of seed biomolecules.

### 3.3. Germination Studies

To assess the germination response to temperature, 100 seeds of each lot were placed on top of the paper, between the paper, and on the sand. The seeds were allowed to germinate under varying temperatures (25, 30, and 35 °C) to determine the optimal temperature for growth in separate germination chambers set at the three different temperatures. To evaluate the effect of temperature on seedling growth, seedlings were randomly collected seven days after germination, and germination counts were conducted. The standard calculation for germination was used as per the ISTA protocol [87]:

$$Germination(\%)=\frac{Number\;of\;normal\;seedlings}{Total\;number\;of\;seeds\;used}\times 100$$

The fresh and dry weight of seedlings was measured. The samples were placed in an oven at 80 °C for 72 h for dry weight. Root and shoot lengths were also measured on the seventh day for the final count. Vigor index I and vigor index II were calculated as follows:

Seedling vigor index I = Standard germination (%) × Full seedling length (cm);

Seedling vigor index II = Standard germination (%) × Seedling dry weight (mg).

### 3.4. Seed Viability Test

Tetrazolium (2,3,5-triphenyl tetrazolium chloride) solutions with concentrations of 0.1, 0.5, 1.0, and 2.0% and three temperatures of 25, 30, and 35 °C were used to assess the viability of moringa seeds. Two hundred seeds in three replications of 50 seeds were tested. The seeds were soaked in distilled water overnight to enhance enzyme activation, progression of embryo activities, and staining and assessment characteristics. Moringa seeds are dicots; hence, the seed coat and membrane below the seed coat were removed before soaking the seeds in tetrazolium solution. Then, the seeds were soaked in solutions of different concentrations at various temperatures, and the staining was assessed every hour until the completion of the process. The seed viability percentage was calculated based on completely stained seeds. The seeds in which no color was observed were counted as dead seeds.

### 3.5. Total Phenolic Compounds

Polyphenols were extracted from finely grounded samples (1 g) by incubating in 1% HCl/methanol for two hours with shaking, followed by centrifugation. Total phenols were calculated using gallic acid as a reference, and the data are represented as mg gallic acid equivalents/g sample (GAE) [88]. Total flavonoids were determined as per the method described using catechin as a standard [89]. Chlorogenic acid was the standard to measure o-dihydroxy phenols [90]. Rutin was used as a reference to compute flavanol content [91].

### 3.6. In Vitro Antioxidant Assays

Fresh plant tissue weighing 100 mg was homogenized in liquid nitrogen (−196 °C) and then extracted using cold 80% ethanol. The ethanolic extract was combined with the ferric-reducing antioxidant power (FRAP) reagent [92]. Trolox was employed as a reference. For the DPPH assay, an appropriate volume of DPPH solution prepared in methanol was applied to the moringa extract. After incubation at room temperature for 40 min, absorbance was measured at 515 nm. The activity was expressed in mg Trolox equivalent/g sample (TE) [92]. ABTS was assessed using the assay described in [93] with minor alterations. A mixture of 2.45 mM potassium persulfate solution and 7 mM ABTS solution was used to prepare an ABTS radical cation solution, which was then incubated for 16 h in a dark room. Then, absorbance was measured at 734 nm.

### 3.7. UPLC/ESI/QTOF-MS of Moringa Extracts

The phenolic profiles of fresh *Moringa oleifera* seedlings were assessed using ultra-high-performance liquid chromatography instrumentation connected to a PDA and combined with electrospray ionization quadruple-pole time-of-flight mass spectrometry equipment from Agilent Technologies (UHPLC/ESI/QTOF-MS, Micromass, Newcastle, UK) [47]. To acquire maximum signals for polyphenols, the optimized TQD tuning parameters were as follows: capillary voltage, 2.0 kV; cone voltage, 36 V; source temperature, 120 °C; desolvating temperature, 500 °C; source desolvating gas flow, 1000 L/h; and cone gas flow, 50 L/h. The flow rate was 0.25 mL/min and the injection volume was 5 μL. Mass spectrometry data were obtained using electrospray ionization in positive ionization mode. The range of the entire scan mass was from m/z 100 to 1200. High-purity nitrogen was utilized as a desolvation, cone, and collision gas. Finally, 1 µL of the solution was injected into the UPLC-ESI-MS/MS system for analysis. Data were obtained using MassLynx software (version 4.1) and processed using the TargetLynx program.

### 3.8. Polyamines

For the quantification of polyamines, 1 g of fresh tissue was first homogenized in 5 mL of cold 5% perchloric acid [94]. After 30 min of incubation, the supernatant following centrifugation is moringa extract, and it was stored at −20 °C for further benzoylation of polyamines. A mixture of 500 µL of moringa extract, 1 mL of 2N NaOH, and 10 µL of 99% benzoyl chloride was used for benzoylation. The reaction was then saturated with 2 mL of NaCl. Diethyl ether was used to extract the benzoylated polyamines, which were then separated using an HPLC (Shimadzu, SCL 10AP VT, Kyoto 604-8511, Japan) equipped with a C_18_ ODS 2 analytical column that had already been pre-equilibrated and operated isocratically for 40 min with acetonitrile/water (50:50) as the mobile phase, with an injection volume of 10 µL. The absorbance was read at 224 nm in a UV detector and expressed as nmol g^−1^ fresh weight. The integration zones obtained for the samples and the standards were compared to determine the concentration of polyamines in the eluent.

### 3.9. Antioxidative Enzymes

The enzymatic extract was homogenized in the fresh sample in 100 mM sodium phosphate buffer, pH 7.5, and 1 mM EDTA.

Superoxide dismutase was assayed by measuring its ability to inhibit the photochemical reduction of nitro-blue tetrazolium (NBT) [95]. The 3.0 mL reaction mixture contained 2.5 mL of 60 mM Tris-HCl (pH 7.8) and 0.1 mL each of 420 mM L-methionine, 1.80 mM NBT, 90 µM riboflavin, 3.0 mM EDTA, and enzyme extract. The tubes were thoroughly shaken and placed 30 cm below a light source of three 20 W fluorescent lamps (Philips Global Business Services LLP, Calcutta, India). The absorbance was recorded at 560 nm. One enzyme unit was defined as the amount of enzyme required to inhibit the photoreduction of 1 µmol of NBT, which was expressed as U g^−1^ fresh weight and converted to U mg protein^−1^ by estimating the sample total soluble proteins. The percentage inhibition was calculated following the formula of Asada et al. [96]. Percentage inhibition = V − v/v × 100, where V = rate of assay reaction in the absence of SOD and v = rate of assay reaction in the presence of SOD. Catalase activity was measured according to the method of Aebi [97]. The reaction mixture (1.0 mL) consisted of 0.5 mL of 0.2 M phosphate buffer (pH 7.0), 0.4 mL of 0.2 M hydrogen peroxide, and 0.1 mL of enzyme extract. The reaction mixture was incubated at 37 °C. The reaction was terminated by adding a 3 mL mixture of 5% (*w*/*v*) potassium dichromate and glacial acetic acid (1:3 *v*/*v*) and heating for 10 min. The absorbance of the test sample and control was measured at 570 nm. The catalase activity (CAT; EC 1.11.1.6) was measured by observing the dissociation of H_2_O_2_ at a wavelength of 240 nm. Peroxidase was assayed according to the method of Shannon et al. [98]. The reaction mixture, with a final volume of 2.9 mL, contained 2.5 mL of 50 mM phosphate buffer (pH 6.5), 0.1 mL of 0.5% hydrogen peroxide, 0.1 mL of 0.2% *o*-dianisidine, and 50 µL of enzyme extract. The change in absorbance was recorded at 430 nm for 3 min at 15 s intervals. One unit of peroxidase represents a change of 1.0 O.D. in 1 min. The enzyme activity was measured using the method of Nakano and Asada [96], which is based on the spectrophotometric monitoring of ascorbic acid oxidation. The reaction mixture for the APX activity assay consisted of 2.5 mL of 0.1 M phosphate buffer (pH 7.0), 0.25 mL of 1.2 mM EDTA, 0.1 mL of 15 mM ascorbic acid, and 0.1 mL of enzyme extract. The reaction was initiated by adding 0.05 mL of 35 mM H_2_O_2_. One enzyme unit is defined as the enzyme required to oxidize 1 nmol of ascorbic acid per min at 290 nm. The glutathione peroxidase activity was determined using a method based on the continuous regeneration of oxidized glutathione produced by the action of glutathione peroxidase [99]. The reaction mixture consisted of 2.0 mL of 0.1 M phosphate buffer (pH 8.0), 0.1 mL of 1 M NaCl, 75 µL of 10 mM EDTA, 0.3 mL of 10 mM GSH, 0.15 mL of 8 mM NADPH, 25 µL of GR (100 U mL^−1^), 0.25 mL of enzyme extract, and 0.1 mL of 25 mM H_2_O_2_. The kinetic changes in the absorbance were measured by the decrease in the absorbance at 340 nm for 15 min at 30 °C. The GPX activity was measured by evaluating the non-enzymatic oxidation of NADPH with an extinction coefficient [ε = 6.22 mM^−1^ cm^−1^]. The reaction mixture consisted of 2 mL of 0.1 M phosphate buffer (pH 7.5), 0.1 mL of 5 mM oxidized glutathione (GSSG), 0.1 mL of 3.5 mM NADPH, and 0.1 mL of enzyme extract for a final volume of 2.3 mL. The decrease in absorbance at 340 nm due to the oxidation of NADPH was monitored. Non-enzymatic oxidation of NADPH was recorded and subtracted. One enzyme unit was defined as the amount of enzyme required to oxidize 0.1 µmol of NADPH oxidized per min. According to Lowry et al. [100], 100 mg of fresh tissue was homogenized to extract the soluble proteins for specific enzyme activity.

### 3.10. Antioxidant Molecules

Tissue (1 g) was homogenized in 5 mL of 5% (*w*/*v*) meta-phosphoric acid in glacial acetic acid and centrifuged at 10,000× *g* for 25 min [101]. The supernatant obtained was used for the estimation of ascorbic acid content. A total of 1.9 mL of distilled water, 1 mL of 2% 2,4-dinitrophenyl hydrazine (in an acidic medium), and one drop of 10% thiourea (in 70% ethanol) were added to 0.1 mL of diluted aliquot, thoroughly mixed, and incubated in a water bath for 15 min. Then, the mixture was cooled to room temperature. Absorbance was then read at 530 nm. The ascorbic acid standard curve (10–100 g) was used to calculate the ascorbic acid concentration at 530 nm. The carotenoid content of the leaf was determined following extraction in DMSO, with the absorbance measured at 480, 645, and 663 nm [102].

### 3.11. Compatible Osmolytes

Proline content in the sample was determined by first homogenizing the fresh sample in 3% aqueous sulfosalicylic acid and centrifuging at 10,000 rpm for 15 min [103]. A total of 2 mL of acid ninhydrin reagent (prepared by adding 1.25 g ninhydrin, 30 mL glacial acetic acid, and 20 mL 6 M phosphoric acid) and glacial acetic acid were added to the supernatant. The mixture was heated in a boiling water bath for one hour for color development. The reaction was stopped by placing the tubes holding the sample in an ice bath. Toluene was added to the reaction mixture, stirring for 20–30 s. The toluene layer was separated and then warmed to room temperature. The chromophore containing toluene was aspirated from the aqueous phase and O.D. was recorded at 520 nm (red color intensity) using toluene as a blank. The standard curve was prepared with proline (0–0.25 µmol/mL). Glycine betaine (GB) estimation was carried out using 500 mg of finely powdered sample [104]. Finely powdered plant material (0.5 g) was mechanically shaken with 20 mL of deionized water for 48 h at 25 °C and the extracts were diluted 1:1 with 2 N sulfuric acid. Cold potassium iodide/iodine reagent (0.2 mL) was added and the mixture was gently mixed by vortex. The samples were stored at 0–4 °C for 16 h. After that, samples were centrifuged at 10,000× *g* for 15 min at 0 °C. The supernatant was dissolved in 9 mL of 1,2-dichloro ethane (reagent grade). Vigorous vortex mixing was carried out to effect complete solubility in developing solvent. After 2.0–2.5 h, the absorbance was measured at 365 nm using a UV–visible spectrophotometer. GB reference standards (50–200 mg mL^−1^) were prepared in 2 N sulfuric acid.

### 3.12. Nonstructural Carbohydrates

Here, 10 mL of 80% (v/v) ethanol was added to 0.10 g of freeze-dried material and the mixture was homogenized for 1 min. The soluble sugars were then extracted by incubating the mixture in a water bath maintained at 80 °C for 1 h. The mixture was then held overnight at 4 °C. The supernatant was removed after centrifugation at 10,000× *g* for 20 min at 4 °C and then dried in a vacuum concentrator [105]. The concentration of each sugar was calculated for individual sugars by comparison to sugar standards.

### 3.13. Semi-Quantitative Gene Expression Analysis

The Qiagen Plant Total RNA Miniprep Kit was used to extract total RNA from fresh tissue (Qiagen Inc., Germantown, MD, USA). The RNA quantity was checked using Picodrop (Picodrop Ltd., Cambridge, UK). The amount of total RNA was calculated at 260 nm, and an A260:A280 ratio in the range of 1.8–2.0 was selected for further investigation. An iScript cDNA synthesis kit (Bio-Rad Laboratories, Inc., Pleasanton, CA, USA) was used for single-stranded cDNA synthesis. Five technical triplicates and three biological duplicates were used in the experiment. Then, 0.2 M gene-specific forward primers and reverse primers (0.5 µL each), Taqman-polymerase master mix (2X), reaction buffer (12 µL), and nuclease-free water (NFW) (10 µL), resulting in a reaction volume of 25 µL, were used in the semi-quantitative RT-qPCR analysis. The PCR reaction in the thermocycler (Applied Biosystems VeritiPro Thermal Cycler, Applied Biosystems, Thermo Fisher Scientific, Waltham, MA, USA) was carried out as follows: (1) denaturation at 94 °C for 3 min; (2) 30 cycles of 94 °C for 30 s, annealing at 57 °C for 45 s, and extension at 72 °C for 10 min. The PCR products were examined on a 1.5% agarose gel. The band intensity was quantified in ImageJ 1.51 K (http://imagej.nih.gov/ij, accessed on 21 May 2022) densitometric software.

#### Primer Designing

Primers for the candidate genes were designed using Primer-BLAST v3 software from NCBI, namely, Cu/Zn superoxide dismutase 1 (*Cu/Zn-SOD*), ascorbate peroxidase (*APX*), GDP-L-galactose phosphorylase (*GGP*), and trehalose-6-phosphate synthase (*TPS*) (Table 5), which were then custom synthesized by Sigma. To normalize the data, glyceraldehyde-3-phosphate dehydrogenase (*GAPDH*) was used as the internal reference gene.

### 3.14. Statistical Analysis

Data are presented as mean ± SD. Two-way ANOVA was performed to assess the importance of the significant factors (sample tissue, temperature, and their interaction with growth indices). Significant variations in the parameter were determined using Tukey’s test for a 5% significance level. The statistical analysis was performed using SPSS v23.0 software (SPSS for Windows, Chicago, IL, USA).

## 4. Conclusions

In conclusion, this study demonstrates that moringa seeds’ nutritional and bioactive quality can be effectively enhanced through specific germination conditions, with a degree of improvement depending on germination temperature and time. Germination at 30 °C leads to a dominance of polyphenols, high antioxidant capacity, and functional activity of phytochemicals. Moreover, aqueous extracts of germinated moringa exhibit strong scavenging effects, including DPPH ABTS and lipid peroxidation inhibition, especially in leaf tissue. Furthermore, UPLC/QTOF-MS analysis reveals the presence of various polyphenolic compounds in ethanolic extracts of moringa seedlings. The expression of *Cu/Zn*-*SOD*, *APX*, *GGP*, and *TPS* genes at 30 °C suggests that the PKM-1 variety possesses desirable genetic traits for temperature resistance. Based on these findings, optimized germinated *M. oleifera* seedlings hold great potential as a novel source of natural antioxidants and α-glucosidase inhibitors, offering promising applications in the pharmaceutical industry and the development of functional foods.

## Figures and Tables

**Table 1:** Polyphenolic composition of *Moringa oleifera* grown under different temperatures.

Sample Tissue	Temperature	TP (mg GAE/100 DW g)	TF (mg RE/100 DW g)	Flavanols (mg/100g DW)	*o*-HP (mg/100 g DW)
Leaves	25 °C	844.21 ± 8.92 ^b^	39.24 ± 0.22 ^b^	0.195 ± 0.22 ^b^	0.874 ± 0.23 ^b^
	30 °C	940.73 ± 9.7 ^a^	45.34 ± 0.35 ^a^	0.292 ± 0.052 ^a^	0.924 ± 0.21 ^a^
	35 °C	652.49 ± 4.8 ^c^	17.23 ± 0.11 ^c^	0.117 ± 0.23 ^c^	0.243 ± 0.11 ^c^
Whole seedling	25 °C	615.26 ± 3.8 ^b^	26.87± 0.23 ^b^	0.211 ± 0.23 ^b^	0.854 ± 0.25 ^b^
	30 °C	785.26 ± 6.4 ^a^	35.24 ± 0.29 ^a^	0.255 ± 0.32 ^a^	0.875 ± 0.29 ^a^
	35 °C	524.36 ± 4.6 ^c^	18.69 ± 0.12 ^c^	0.184 ± 0.36 ^c^	0.714 ± 0.22 ^c^
Roots	25 °C	154.26 ± 1.4 ^b^	20.15 ± 0.12 ^b^	0.194 ± 0.16 ^b^	0.711 ± 0.23 ^b^
	30 °C	191.24 ± 1.7 ^a^	26.25 ± 0.13 ^a^	0.212 ± 0.15 ^a^	0.741 ± 0.31 ^a^
	35 °C	142.24 ± 1.1 ^c^	14.25 ± 3.10 ^c^	0.165 ± 0.11 ^c^	0.658 ± 0.26 ^c^
Seeds	25 °C	185.23 ± 2.6 ^b^	22.12 ± 0.22 ^b^	0.225 ± 0.22 ^b^	0.789 ± 0.31 ^b^
	30 °C	221.18 ± 3.1 ^a^	32.21 ± 0.13 ^a^	0.235 ± 0.26 ^a^	0.815 ± 0.36 ^a^
	35 °C	165.28 ± 2.7 ^c^	16.54 ± 0.14 ^c^	0.174 ± 0.21 ^c^	0.615 ± 0.28 ^c^

Different superscript letters within a column indicate significant differences (two-way ANOVA, post hoc Tukey’s test, *p* ≤ 0.05). GAE (gallic acid equivalents), DW (dry weight basis), *o*-HP (*o*-hydroxyphenols), TP (total phenols), RE (rutin equivalents), and TF (total flavonoids).

**Table 2:** Antioxidant activity of *M. oleifera* extracts.

Sample Tissue	Temperature	IC_50_ DPPH (µg/mL)	IC_50_ ABTS (µg/mL)	IC_50_ FRAP (µg/mL)
Leaves	25 °C	6.34 ± 0.22 ^b^	14.24 ± 0.22 ^b^	125.2 ± 1.22 ^b^
	30 °C	2.39 ± 0.11 ^a^	10.34 ± 0.35 ^a^	117.4 ± 1.02 ^a^
	35 °C	9.77 ± 0.32 ^c^	19.23 ± 0.11 ^c^	257.5 ± 1.26 ^c^
Whole seedling	25 °C	7.26 ± 0.8 ^b^	12.87 ± 0.23 ^b^	211.5 ± 1.23 ^b^
	30 °C	4.56 ± 0.4 ^a^	12.24 ± 0.29 ^a^	184.3 ± 1.12 ^a^
	35 °C	12.36 ± 0.6 ^c^	20.69 ± 0.12 ^c^	235.4 ± 1.36 ^c^
Roots	25 °C	11.26 ± 0.4 ^b^	15.15 ± 0.12 ^b^	194.2 ± 1.16 ^b^
	30 °C	9.24 ± 0.7 ^a^	13.25 ± 0.13 ^a^	165.8 ± 1.10 ^a^
	35 °C	15.24 ± 0.1 ^c^	24.25 ± 0.15 ^c^	212.5 ± 1.21 ^c^
Seeds	25 °C	13.23 ± 0.6 ^b^	18.12 ± 0.22 ^b^	225.1 ± 1.22 ^b^
	30 °C	10.18 ± 0.1 ^a^	15.21 ± 0.13 ^a^	174.2 ± 1.16 ^a^
	35 °C	21.28 ± 0.7 ^c^	29.54 ± 0.14 ^c^	235.6 ± 1.31 ^c^

Different superscript letters within a column indicate significant differences (two-way ANOVA, post hoc Tukey’s test, *p* ≤ 0.05).

**Table 3:** UPLC/QTOF-MS data of the polyphenolic compounds in *Moringa oleifera* seedlings.

Rt (min)	Neutral Mass (Da)	Observed Mass m/z	Mass Error (ppm)	Adducts	Phenolic Compound
0.82	558.137	617.149	−3.7	+CH3COO	(+)-Gallocatechin hexaacetate
0.83	606.195	665.210	2.2	+CH3COO	5,7-Dimethoxy-4′-hydroxyflavone-4′-*O*-alpha-L-rhamnose(1-->2)-beta-D-glucoside
0.84	652.200	711.214	−0.7	+CH3COO	Nevadensin-7-*O*-[α-L-rhamnosyl(1-->6)]-beta-D-glucoside
0.85	786.258	845.277	5.3	+CH3COO	Echinacoside
0.86	150.053	195.050	−5.8	+HCOO	Ribose
0.87	342.110	341.105	4.6	−H, +HCOO	4′,5,6,7-Tetramethoxy-flavone
0.87	342.116	387.112	−5.8	+HCOO	Maltose
0.88	720.211	719.197	−9.7	−H	Chelidimerine
0.90	180.063	179.055	−4.1	−H	Glucose
0.95	88.016	133.014	−2.9	+HCOO	Pyruvic acid
1.04	330.095	375.092	−4.3	+HCOO	Vanillic acid-4-*O*-β-D-glucopyranoside
1.10	129.043	128.035	−1.2	−H	5-Oxoproline
1.18	332.074	331.069	5.7	−H	1-Galloyl-glucose
1.26	425.045	424.036	−5.2	−H	Glucosinalbin
1.54	138.032	137.026	−6.4	−H	Protocatechuic aldehyde
1.67	466.111	511.109	−0.3	+HCOO	Taxifolin-3-*O*-glucoside
1.98	466.111	511.112	4.7	+HCOO	(2*R*,3*R*)-Taxifolin-3′-*O*-beta-D-glucopyranside
2.16	449.108	508.122	−0.7	+CH3COO	Cyanidin-3-glucoside
2.24	452.132	497.132	3.4	+HCOO	Catechin-7-*O*-β-D-glucopyranoside
2.35	304.058	349.057	0.4	+HCOO	(2*R*,3*R*)-3,5,7,2′,6′-Pentahydroxyvflavanone
2.44	180.042	225.042	5.6	+HCOO	3,4-Dihydroxycinnamic acid
2.58	402.153	401.143	−5.7	−H, +HCOO	Benzyl alcohol xylopyranosyl-(1-->6)-glucopyranoside
2.60	330.095	329.089	3.6	−H	Vanillic acid-4-*O*-β-D-glucopyranoside
2.69	326.100	385.115	2.8	+CH3COO	4-*O*-β-D-Glucopyranosyl-*cis*-cinnamic acid
3.66	312.121	357.120	3.7	+HCOO	Piscidic aciddiethyl ester
4.13	326.137	371.135	0.4	+HCOO	Eugenyl glucoside
4.29	396.142	395.135	1.1	−H	Propyl chlorogenate
5.96	448.137	447.131	1.9	−H	(2*S*)-5,7-Dihydroxy-6-methoxy-flavanone-7-*O*-β-D glucopyranoside
7.02	316.261	315.252	−7.2	−H	Dihydroxy stearic acid
8.37	598.314	597.305	−3.3	−H	Daturametelin H
9.44	282.256	281.248	−2.3	−H	(*E*)-9-Octadecenoic acid
10.72	414.386	473.403	6	+CH3COO	24-Ethyl cholesterol
12.25	326.188	325.15	9.7	−H	Pregna-4,16-diene-3,12,20-trione

**Table 4:** Profiles of different polyamines (Put, Spm, and Spd) in moringa seedlings under different temperatures.

Sample Tissue	Temperature	Put *	Spd *	Spm *
Leaves	25 °C	382.47 ± 1.8 ^c^	314.21 ± 1.9 ^b^	39.64 ± 0.34 ^d^
	30 °C	457.64 ± 2.3 ^d^	357.95 ± 2.0 ^a^	45.16 ± 0.64 ^e^
	35 °C	336.46 ± 1.4 ^b^	246.84 ± 1.1 ^e^	25.58 ± 0.62 ^c^
Whole seedling	25 °C	297.69 ± 1.5 ^d^	271.21 ± 1.2 ^d^	23.98 ± 0.25 ^e^
	30 °C	364.12 ± 1.7 ^e^	287.46 ± 1.4 ^c^	26.57 ± 0.62 ^e^
	35 °C	270.95 ± 1.2^a^	164.28 ± 1.3 ^b^	18.59 ± 0.61 ^d^
Roots	25 °C	155.69 ± 1.3^a^	134.21 ± 1.1 ^d^	15.53 ± 0.98 ^a^
	30 °C	191.85 ± 1.2 ^b^	141.49 ± 1.2 ^c^	20.64 ± 0.21 ^d^
	35 °C	233.54 ± 1.4 ^c^	123.65 ± 0.68 ^e^	10.86 ± 0.37 ^b^
Seeds	25 °C	131.42 ± 1.1 ^c^	99.25 ± 0.95 ^d^	6.38 ± 0.23 ^a^
	30 °C	144.13 ± 1.3 ^d^	117.27 ± 1.2^c^	10.52 ± 0.16 ^d^
	35 °C	113.55 ± 0.43 ^e^	88.64 ± 0.67 ^e^	4.59 ± 0.03 ^a^

* nmol g^−1^ FW; FW: fresh weight; Put: putrescine; Spm: spermine; Spd: spermidine. Different superscript letters within a column indicate significant differences (two-way ANOVA, post hoc Tukey’s test, *p* ≤ 0.05).

**Table 5:** List of primers used in the study.

Genes	Accession Number	Primers	Sequence (5′-3′/3′-5′)
*Cu/Zn-SOD*	JQ284380.1	Forward	ATCCTGCTGGCAAAGAACAT
		Reverse	ATCTGCATGGACAACGACAG
*GGP*	AB924665.1	Forward	CAAGCTCTTGGGGAAGTGAG
		Reverse	TCACCGAGCTCTGTTCATTG
*TPS*	MG736641.1	Forward	GGCCATTACAATGCCAGAGT
		Reverse	ACAATAGGTTCCACGGCAAG
*APX*	JQ284377.1	Forward	TTGAGGGTCGTCTTCCTGAT
		Reverse	GCAAAAGCCCTTCCTTCTCT
*GAPDH*	JQ764560.1	Forward	TGCTAGCTGCACTACCAACTG
		Reverse	TCCTGTGCTGCTAGGAATGA

## Data Availability

Not applicable.

## Supplementary Materials

The following supporting information can be downloaded at https://www.mdpi.com/article/10.3390/plants12163010/s1, Figure S1. Effect of temperature on the germination studies of moringa seedlings at the early development stage; Figure S2. Chemical structures of the polyamines discussed in this study; Figure S3. HPLC chromatogram of benzoylated polyamine: (a) HPLC chromatogram of benzoylated polyamine standards, Put, Spd, and Spm, at a concentration of 0.05 mM; (b) identification of polyamines in moringa extracts with each of Put, Spd, and Spm.
