# Supplementary material for: Probing the Phytochemical Composition and Antioxidant Activity of Moringa oleifera under Ideal Germination Conditions
Source: Plants (Basel). 2023 Aug 21;12(16):3010. doi: 10.3390/plants12163010 (PMC10459117; doi:10.3390/plants12163010)
Supplement: Supplementary file 1 [file plants-12-03010-s001.zip › plants-2445170-supplementary.pdf]

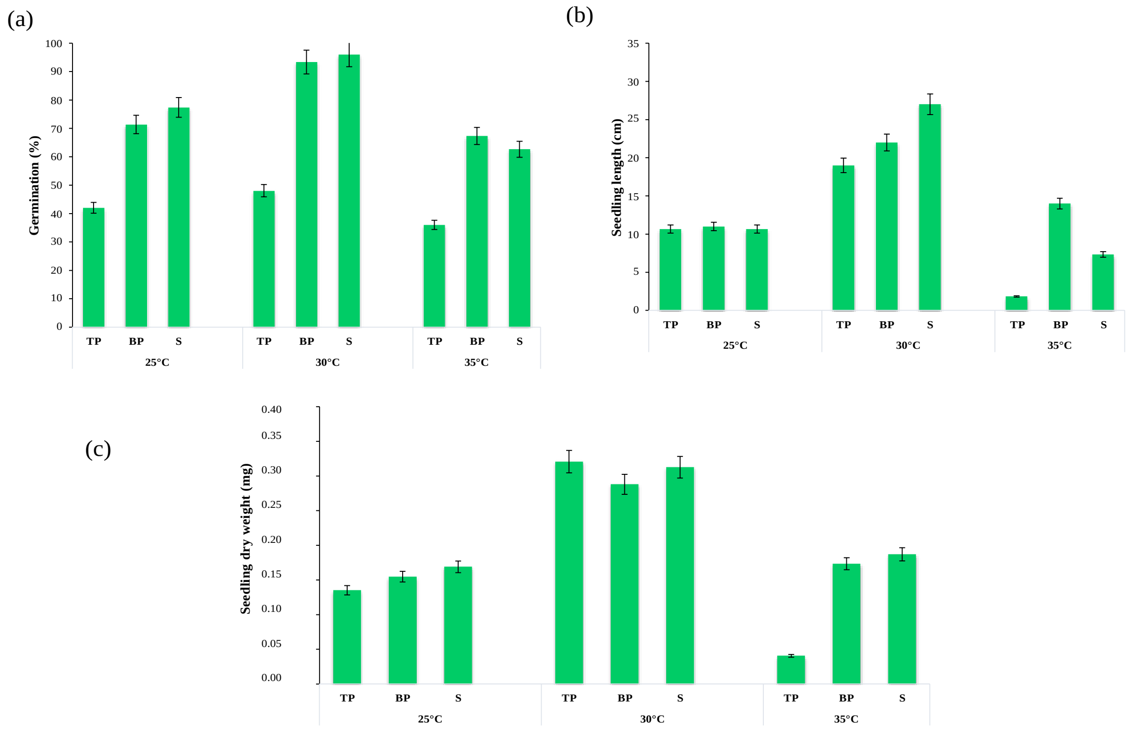

Supplementary Figure S1. Effect of temperature on germination studies of Moringa seedlings at the early growth stage. (a) Standard germination (b) seedling length; (c) dry weight; (d) vigour index-I; (e) vigour index-II. Data are expressed as Mean±SD. Values are presented on a fresh weight (FW) basis.

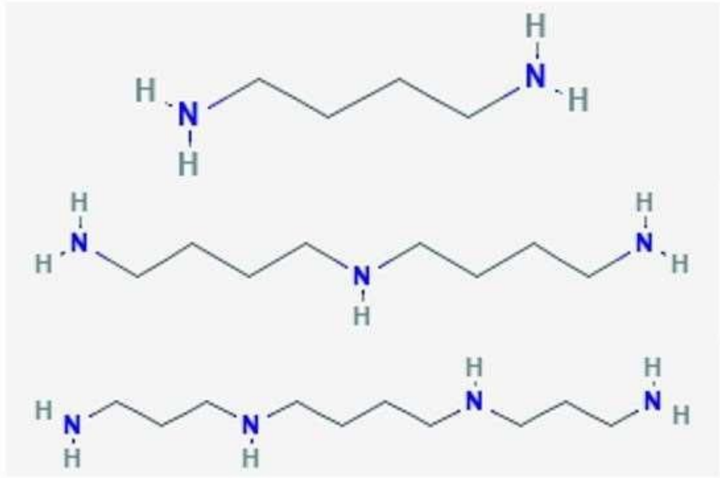

Put

Spd

Spm

Supplementary Figure S2. Chemical structures of the polyamines discussed in this study.

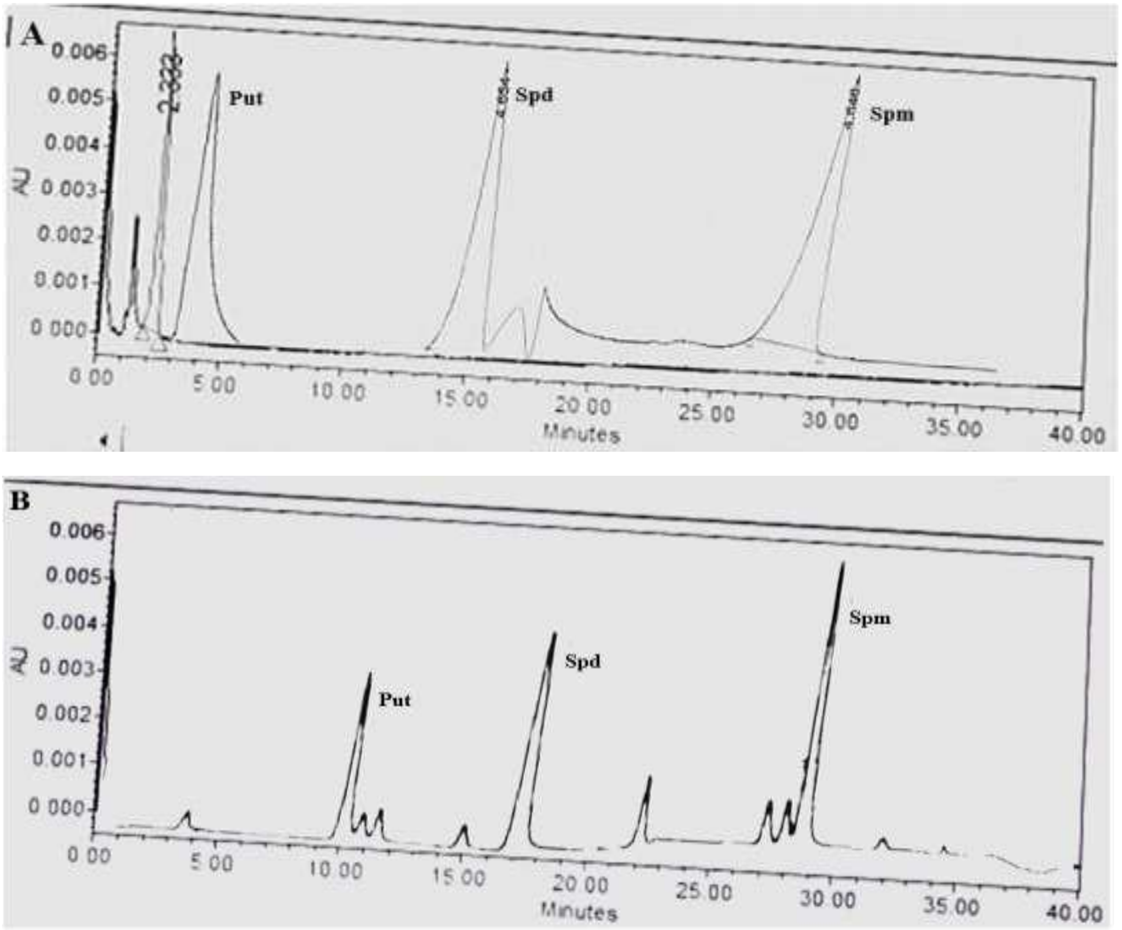

Supplementary Figure S3. HPLC chromatogram of benzoylated polyamine (a) HPLC chromatogram of benzoylated polyamine standards; Put, Spd, and Spm at 0.05mM concentration (b) Identification of polyamines in Moringa extracts with each of each Put, Spd, and Spm.
